# The Potential of Class II Bacteriocins to Modify Gut Microbiota to Improve Host Health

**DOI:** 10.1371/journal.pone.0164036

**Published:** 2016-10-03

**Authors:** Özgün C. O. Umu, Christine Bäuerl, Marije Oostindjer, Phillip B. Pope, Pablo E. Hernández, Gaspar Pérez-Martínez, Dzung B. Diep

**Affiliations:** 1 Department of Chemistry, Biotechnology and Food Science, Norwegian University of Life Sciences (NMBU), Ås, Norway; 2 Departamento de Biotecnología, Instituto de Agroquímica y Tecnología de Alimentos (IATA), Consejo Superior de Investigaciones Científicas (CSIC), Valencia, Spain; 3 Departamento de Nutrición, Bromatología y Tecnología de los Alimentos, Facultad de Veterinaria, Universidad Complutense de Madrid (UCM), Madrid, Spain; Universitat Ulm, GERMANY

## Abstract

Production of bacteriocins is a potential probiotic feature of many lactic acid bacteria (LAB) as it can help prevent the growth of pathogens in gut environments. However, knowledge on bacteriocin producers *in situ* and their function in the gut of healthy animals is still limited. In this study, we investigated five bacteriocin-producing strains of LAB and their isogenic non-producing mutants for probiotic values. The LAB bacteriocins, sakacin A (SakA), pediocin PA-1 (PedPA-1), enterocins P, Q and L50 (enterocins), plantaricins EF and JK (plantaricins) and garvicin ML (GarML), are all class II bacteriocins, but they differ greatly from each other in terms of inhibition spectrum and physicochemical properties. The strains were supplemented to mice through drinking water and changes on the gut microbiota composition were interpreted using 16S rRNA gene analysis. In general, we observed that overall structure of the gut microbiota remained largely unaffected by the treatments. However, at lower taxonomic levels, some transient but advantageous changes were observed. Some potentially problematic bacteria were inhibited (e.g., *Staphylococcus* by enterocins, Enterococcaceae by GarML, and *Clostridium* by plantaricins) and the proportion of LAB was increased in the presence of SakA-, plantaricins- and GarML-producing bacteria. Moreover, the treatment with GarML-producing bacteria co-occurred with decreased triglyceride levels in the host mice. Taken together, our results indicate that several of these bacteriocin producers have potential probiotic properties at diverse levels as they promote favorable changes in the host without major disturbance in gut microbiota, which is important for normal gut functioning.

## Introduction

With more than 1,000 bacterial species residing in the gastrointestinal tract [1], the gut microbiota is expected to have developed means to compete with each other for common resources and strategies to cope with different pressures from the host [2]. Survivors are selected on the basis of several aspects, including the ability to deal with the host diet, colonization resistance, inhibitory agents (e.g., bile salt, defensins) and other host-mediated effects like improved barrier function and altered immune response [3]. Bacteria, the most predominant members of the gut microbiota, use different mechanisms to colonize and persist in the gut. One of these is the production of bacteriocins, which are ribosomally synthesized antimicrobial peptides produced by numerous Gram-negative and Gram-positive bacteria [4]. Most bacteriocins have relatively narrow spectra, normally targeting species or genera closely related to the producers. Others, such as the lactococcal bacteriocin nisin, can have a much wider spectrum, including important pathogenic or problematic species of *Staphylococcus*, *Listeria*, *Enterococcus* and *Streptococcus* [4,5]. A large number of bacteriocins are produced by lactic acid bacteria (LAB), which constitute a diverse group of bacteria that are frequently found in food and feed, as well as being common inhabitants in the gut environment of a great number of animals, including humans. LAB are therefore generally regarded as safe (GRAS) for human consumption and the production of bacteriocins as one of the important probiotic properties. It has been shown that bacteriocins can modulate the host immune system, as well as being able to antagonize opportunists and potential pathogens [6].

Bacteriocins produced by Gram-positive bacteria are classified into two main classes: class I, containing heavily modified (lanthionine-containing) peptides called lantibiotics; and class II, containing non-modified peptides or peptides with minor modifications (such as disulfide bond formation or circularization) [7]. Class II bacteriocins can be divided further into subclasses: class IIa, pediocin-like bacteriocins, which are typically very active against *Listeria* and have a relatively narrow spectrum; class IIb, two-peptide bacteriocins, whose activity is dependent on the synergy between two different peptides; class IIc, circular bacteriocins; and class IId, the miscellaneous group which include all other bacteriocins that do not fit into any of the aforementioned groups [7].

Mice have been successfully used as a model to unravel the connection between gut microbiota and a variety of health issues or environmental factors, such as obesity [8], diet [9,10] and antibiotics [11]. Also in humans, different external and internal factors can cause changes in the composition of gut microbiota. For instance, it is well known that diet composition can affect distinct human enterotypes [12–14] and that the administration of antibiotics causes drastic changes in the gut microbiota [15]. In addition, the microbiota is altered in certain health conditions such as obesity [16–18], a variety of diseases [19] and stress [2]. However, many changes are transient or can be reverted to normal (healthy) conditions sooner or later dependent on the type of treatments [1].

A number of bacteriocins have been studied for their ability to inhibit pathogens in the gut, such as *Salmonella enteritidis* [20], *Listeria monocytogenes* [21], *Clostridium difficile* [22] and *Staphylococcus aureus* [23]. They have also been observed to eliminate multidrug- or vancomycin-resistant enterococci [24,25] as well as influence some bacteria-related disorders such as obesity [26]. Bacteriocins have several advantages over antibiotics in infection treatments because they are more target-specific and avoid killing of commensal and beneficial cells. They also have low or no toxicity toward eukaryotic cells and are active against both pathogens and their derived antibiotic-resistant strains [27]. However, most studies dealing with gut microbiota lack a detailed assessment on how bacteriocins with different properties affect the general composition of the gut microbiota, and their ability to bring about probiotic effects in healthy animals.

In this study, we performed a comparative study to examine the potential probiotic effects of five different bacteriocin producers on the gut microbiota and other host parameters (including blood serum parameters and weight) in healthy mice. Most of the chosen strains are food-associated lactic acid bacteria, which have been isolated from a variety of food products i.e., meat (*Lactobacillus sake* Lb 706), fermented sausage (*Pediococcus acidilactici* 347 and *Enterococcus faecium* L50) and fermented vegetable (*Lactobacillus plantarum* C11B), while *Lactococcus garvieae* DCC43 is a gut-associated strain that has been isolated from Mallard-duck gut. Their bacteriocins have shown great differences in terms of physicochemical properties, amino acid sequence, target specificity and width of inhibitory spectrum that would allow us to affect the gut at different levels and in different directions. To our knowledge, this is the first study where the probiotic values of various bacteriocins and the interplay between bacteriocin producers and gut microbiota are evaluated in healthy subjects.

## Materials and Methods

### Animals and housing conditions

Six to eight weeks old BALB/C female mice were grouped into 11 different cages (1 control cage with n = 10, 5 cages treated with bacteriocin-producing strains with n = 9 and 5 cages treated with their isogenic non-producing strains with n = 9) and mice were ear-labelled for individual tracking. Before treatments, mice were left in cages for about 10 days for adaptation to the environment after they were brought to the facility. All mice had *ad libitum* access to water and feed and their health status was carefully observed during the entire experimental process. The mice were provided by the Animal Production section of Central Service for Experimental Research (SCSIE) in University of Valencia. All animal work and procedures were approved by the institutional Ethics Committee of the CSIC and University of Valencia and performed following the principles of laboratory animal care (as mandatory by European Union Law and 2010/63/EU and Spanish Government RD 53/2013 on the protection of animals used for scientific purposes).

### Experimental design and sampling scheme

Bacteriocin-producing and non-producing (isogenic mutants) strains of LAB were administrated to mice via drinking water (Table 1). The wildtype bacteriocin producers were: *L*. *sake* Lb 706 producing SakA [28], *P*. *acidilactici* 347 producing PedPA-1 [29], *E*. *faecium* L50 producing enterocins P, Q and L50 [30], *L*. *plantarum* C11B producing plantaricins EF and JK [31] and *L*. *garvieae* DCC43 producing GarML [32]. For each bacteriocin system, a pair of bacterial strains was used: a wildtype producer and an isogenic non-producing mutant, except for one isogenic mutant producing fewer bacteriocins compared to the wildtype strain (Table 1). The isogenic mutant strains were used as negative controls for bacteriocin production. *E*. *faecium* L50 is a multi-bacteriocin producer and its mutant strain has reduced bacteriocin production capacity, i.e., still producing enterocin P but not enterocins Q and L50 (Table 1). Therefore, its isogenic mutant strain was used as negative control for the enterocins Q and L50.

**Table 1:** Administrated bacterial strains and bacteriocins.

Bacterial strain	Bacteriocins in treatments	Class of bacteriocins	Stock Culture (cfu/mL)	References
*L*. *sake* Lb 706	Sakacin A (+)	Class IIa	0.16x10^11^	[28]
*L*. *sake* Lb 706B	Sakacin A (-)		0.2x10^11^	
*P*. *acidilactici* 347	Pediocin PA-1 (+)	Class IIa	2.6x10^11^	[29]
*P*. *acidilactici* 347	Pediocin PA-1 (-)		2.5x10^11^	
*E*. *faecium* L50	Enterocins P, Q and L50 (+)	Class IIb (EntL50) Class IId (EntQ)	0.6x10^11^	[30]
*E*. *faecium* L50/14-2	Enterocin P (+) but Q and L50 (-)		0.5x10^11^	[33]
*L*. *plantarum* C11B	Plantaricins EF and JK (+)	Class IIb	2.6x10^11^	[31]
*L*. *plantarum* C11D3	Plantaricins EF and JK (-)		2.3x10^11^	[34]
*L*. *garvieae* DCC43	Garvicin ML (+)	Class IIc	1.7x10^11^	[32]
*L*. *garvieae* DCC43	Garvicin ML (-)		1.7x10^11^	[35]

Bacterial strains were grown overnight in brain-heart infusion (BHI) medium. Cells were harvested by centrifugation and washed twice with phosphate buffered saline (PBS) before being frozen as stock cultures in 15% glycerol in PBS at -80°C. To prepare bacteria-containing drinking water, each frozen culture was thawed and diluted to give 100 ml water containing about 10^9^ cells/mL, which was then given to each cage (Table 1). The drinking water (with or without bacteria) was renewed on daily basis. The bacterial administration was carried out for 15 days followed by another two more weeks with bacteria-free water. For survival assessment, bacterial cells in drinking water were counted by plating just after dilution and after 24h; this was done once during the first week and once during the second week of the bacterial administration regime. All mice from the same cage shared the same water bottle, and water intake was measured daily for each cage. Fecal samples were collected from each mouse once a week during the four-week experiment. The first sample was taken on day 0 (time zero) just before exposure of mice to bacteria-containing drinking water, thus these samples served as base line. The following fecal samples were collected on day 7, day 14, day 21 and day 28 and kept at -80°C until further analysis. Mice were weighed every week on fecal collection day. Blood samples were collected from the facial vein, without anticoagulants, from 4 to 5 randomized mice from each cage on day 15, which was the last day of bacteria administration. Samples were kept on ice after collection and centrifuged at 1,500 g for 10 min, at 4°C, to separate serum which was collected and kept at -80°C prior to analysis. The samples were analyzed for their triglycerides, total cholesterol, HDL and LDL contents with an Olympus AU400 photometric auto analyzer (Olympus, Tokyo) with the reagents provided by the manufacturer.

### LAB counting and bacteriocin activity

Total LAB cells in fecal samples at day 14 were counted for three randomly selected mice per cage. LAB counting and bacteriocin plate assay were performed as follows: Each fecal pellet was dissolved and serially diluted in 0.9% NaCl. Cells (100μL) from each dilution were mixed with 4 mL of MRS soft agar (0.8%), poured onto an MRS agar plate and then covered by another 4 ml of cell-free soft agar (to prevent cells growing on the surface). Plates were incubated anaerobically at 30°C overnight before being covered with another layer of soft agar containing 100-fold diluted overnight culture of a suitable indicator. The plates were again incubated overnight, and total colony forming units (CFUs) and CFUs of bacteriocin producers that formed inhibition zones were scored. The following indicator strains were used: *E*. *faecium* P21 (LMG 2783) for SakA and PedPA-1, *P*. *damnosus* (LMG 3397) for enterocins, *L*. *plantarum* 965 (LMG 2003) for plantaricins, and *L*. *lactis* IL1403 (LMG2705) for GarML.

### DNA extraction

A total of 495 (99 mice x 5 time points) fecal samples were collected during the course of the experiment. DNA from each fecal sample was extracted using Realpure SSS kit (Real Life-Science Solutions, Durviz, Spain) with addition of a bead-beating step. The DNA was quantified using Qubit® fluorometer with Qubit® dsDNA HS Assay Kit (Invitrogen, Eugene, OR, USA). The DNA samples at each time point from the mice sharing same cage were normalized and pooled prior to the amplicon sequencing, giving rise to 55 pooled samples. DNA samples from three randomly selected mice from the control cage at day 0, day 14 and day 28 were also sequenced to observe the individual variation on fecal microbiota over time.

### 16S rRNA gene amplification and sequencing

Library preparation for 16S rRNA gene amplicon sequencing was performed as described in the Illumina 16S metagenomic sequencing library preparation protocol [36]. Briefly, the V3-V4 region of bacterial 16S rRNA gene [37] was amplified using forward and reverse primers with Illumina overhang adaptors, 5’-TCG TCG GCA GCG TCA GAT GTG TAT AAG AGA CAG CCT ACG GGN GGC WGC AG-3’ and 5'-GTC TCG TGG GCT CGG AGA TGT GTA TAA GAG ACA GGA CTA CHV GGG TAT CTA ATC C-3’, respectively. PCR products were cleaned up using AMPure XP beads (Beckman Coulter Genomics, USA). A second PCR was carried out for sample specific dual indexing using the Nextera XT Index kit (Illumina, San Diego, California, USA) that contains index primers with 8-base indices adjacent to the P5 or P7. Cleaning-up of indexing PCR products was performed again using AMPure XP beads (Beckman Coulter Genomics, USA). Purified product concentrations were measured by Qubit using Qubit® dsDNA HS Assay Kit (Invitrogen, Eugene, OR, USA) and quality was checked by gel electrophoresis (1% agarose gel). Libraries were normalized and pooled. The pool was quantified with rt-PCR using PerfeCta NGS library quantification kit for Illumina Sequencing platforms (Quanta BioSciences, Maryland, USA). The quantified pool was denaturated prior to loading of samples into the MiSeq machine. Loading of libraries to the sequencer was performed using MiSeq v3 reagent kit (Illumina, San Diego, California, USA). The fastq files have been deposited in the SRA (Bioproject ID: PRJNA310414 and Accession number: SRP069889).

### Analysis of sequencing data

The raw Illumina reads were filtered and de-multiplexed using the Illumina MiSeq Reporter system software version 2. The paired-end MiSeq reads were processed using UPARSE pipeline [38] implemented in USEARCH [39] (version 7.0.1090). Paired-ends were merged and quality filtering was applied using maximum expected error (maxee) value of 1.0. Sequences were dereplicated, singletons were discarded. Sequences were clustered into OTUs using 97% sequence identity threshold, chimeric sequences were filtered from clustered OTUs using UCHIME [40] and an OTU table was created. OTUs were processed further using Quantitative Insights Into Microbial Ecology (QIIME) version 1.8.0. The representative OTUs were picked and aligned against the Greengenes core set database [41] using PyNAST [42] with a minimum identity of 75%. Taxonomy was assigned to aligned sequences using The Ribosomal Database Project (RDP) classifier program [43] with a confidence of 0.8. The OTU table was subsampled to normalize the sequence number among samples based on the sample with lowest number of sequences. A phylogenic tree was built using Fast Tree [44] from aligned sequences after the filtration step in order to remove highly variable regions and positions that were all gaps. This tree was used to calculate alpha and beta diversities. Rarefaction curves and Shannon indexes were calculated. Unweighted UniFrac distance metrics [45] were generated and principle coordinate analysis (PCoA) was used to visualize the metrics.

### Statistics

The comparisons of Shannon indexes (S2 Table) between the treatments were performed in R using an ANCOVA that considered time as a continuous dependent variable with significance at P < 0.05. The distances between treatments in PCoA plots were compared in QIIME using a two-sided Student’s *t*-test, and the nonparametric p-values were calculated with 1,000 Monte Carlo permutations using Bonferroni correction. Mixed model ANOVA was performed in R for the comparison of weights where mice were random factor and time points (within subject) and treatments (between subjects) were fixed factors in the model. The changes in relative abundance of taxa in treatments were compared to the changes in CON using ANOVA. Relative abundance at day 0 was taken as basis within each treatment and the change corresponding to day 0-relative abundances were compared to the changes in CON mice. The changes on day 7 and day 14 were together considered to represent the bacteria administration (treatment) period, while day 21 and day 28 represented the post-treatment period. P-values smaller than 0.1 were considered as significant for the relative abundance comparisons when the effect size of testing was high (R^2^ > 0.84). Serum parameters were compared pairwise between the cages treated with bacteriocin-producing and non-producing strains with two-sided Student’s *t*-test in R using fdr correction and the plots were generated for the significantly different parameters using STAMP [46]. Pearson correlations (*P* < 0.05) between serum parameters and the relative abundance of OTUs in treatments were calculated using CoNet [47] and visualized using Cytoscape 3.1.1 [48].

## Results

### Water consumption and weight gain of mice

**Fig 1:**
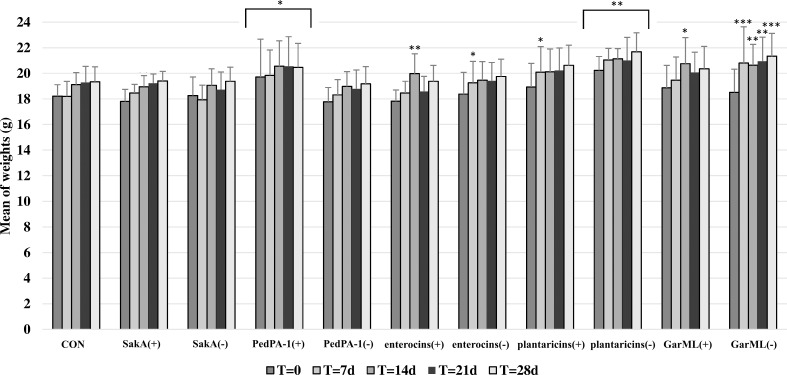
Average weights of mice in the same treatment cage over time. ‘(+)’ represents bacteriocin producer cage while ‘(-)’ represents bacteriocin non-producer cage. Significance degree is represented with stars; p<0.05 with one star (*); p <0.01 with two stars (**); p <0.001 with three stars (***).

The drinking water was refreshed every day and live bacterial cells were kept between 10^8^ to 10^9^ cells per ml for bacteria administration to each cage. The water consumption in all cages was similar to that in the control cage (CON, without bacteria); i.e., mice consuming daily on average 29 ml/cage or 3.2 ml/mouse. In general, most of the mice gained weight in a relatively normal fashion during the course of the 4-week experiment, with an average initial weight at 18.6 g and final weight at 20 g. While mice in most cages gained weight in a fashion comparable to control mice, mice treated with PedPA-1(+) and plantaricins(-) appeared to have higher weight gain, see Fig 1.

### Total LAB and LAB bacteriocin-producers in fecal samples

LAB normally constitute a sizable group in the gastrointestinal tract. We enumerated this group of bacteria in fecal samples to examine how the bacteriocin-positive treatments affect this group, compared to the bacteriocin-negative (isogenic) cages. Using conditions selective for most LAB (MRS plates and anaerobic condition, growth at 30°C), the counts of total LAB were around 10^8^ cfu/g feces for the randomly selected mice from most of the cages (n = 3 from each cage). However, the number of LAB in fecal samples was significantly higher in some bacteriocin positive cages. This is the case for mice treated with the producers of SakA (2-fold, *P* < 0.01), plantaricins (3-fold, *P* < 0.01) and GarML (2-fold, *P* < 0.01) compared to their bacteriocin negative cages (S1 Table).

**Fig 2:**
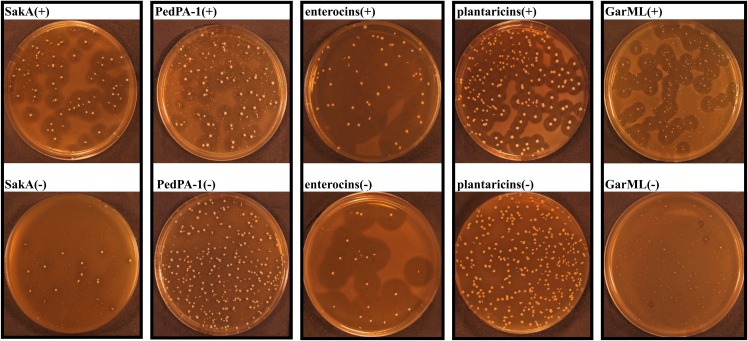
Bacteriocin-producing LAB in the fecal samples of mice at the end of the treatment period. Representative plates from bacteriocin activity assay were shown for each treatment. ‘(+)’ represents bacteriocin producer treatment, while ‘(-)’ represents isogenic non-producer treatment. The clear zones around the colonies indicate bacteriocin production. The indicator strains used in the assay are: *E*. *faecium* P21 (LMG 2783) for SakA and PedPA-1, *P*. *damnosus* (LMG 3397) for enterocins, *L*. *plantarum* 965 (LMG 2003) for plantaricins, and *L*. *lactis* IL1403 (LMG2705) for GarML treatments.

In addition to total LAB enumeration, bacteriocin-producing colonies were counted in the same assay using indicator bacteria that are sensitive to administrated bacteriocins (Fig 2). The portions of bacteriocin producers (tested against the specified indicator strains) among all LAB in bacteriocin positive cages were 25% in SakA(+), 5% in PedPA-1(+), 89% in enterocins(+), 89% in plantaricins(+) and 18% in GarML(+) cages. Interestingly, there were no bacteriocin-producing colonies in the samples from the bacteriocin negative cages, except the cage treated with the enterocins isogenic mutant strain that has lost the genes (by plasmid curing) for the production of enterocin Q and L50, but not the genes for the production of enterocin P (71% bacteriocin producing colonies) (Fig 2).

Moreover, sequencing analysis of the fecal samples showed that the relative abundances of OTUs assigned to *Pediococcus* (*P*. *acidilactici* particularly), *Lactococcus* (*L*. *garvieae* particularly), and unclassified Enterococcaceae, were significantly higher in the cages treated with PedPA-1(+) and (-), GarML(+) and enterocins(+) respectively (S1 Fig). These increases are expected because the bacteriocin producers belong to these bacterial groups. However, *Lactobacillus* had many OTUs assigned; therefore, this effect could not be observed for SakA and plantaricins cages. These results imply that bacteriocin production renders the producers more capable to establish growth in the gut environment.

### Community structure of fecal bacteria

The fecal bacterial community structure was assessed using 16S rRNA gene analysis. The inter-individual variations within the cages were converged by the pooling of DNA samples from mice in the same cage at each time point. Therefore, our results represent the average of many mice as a group within each cage. Nearly 4.8 million merged and quality filtered sequences were obtained. In total 1,168 OTUs were acquired for all samples after chimera filtering. The community of each sample was subsampled, giving about 15,800 sequences/sample. Rarefaction curves indicated that the diversity of bacterial communities was not significantly affected by any of the treatments (S2 Table).

**Fig 3:**
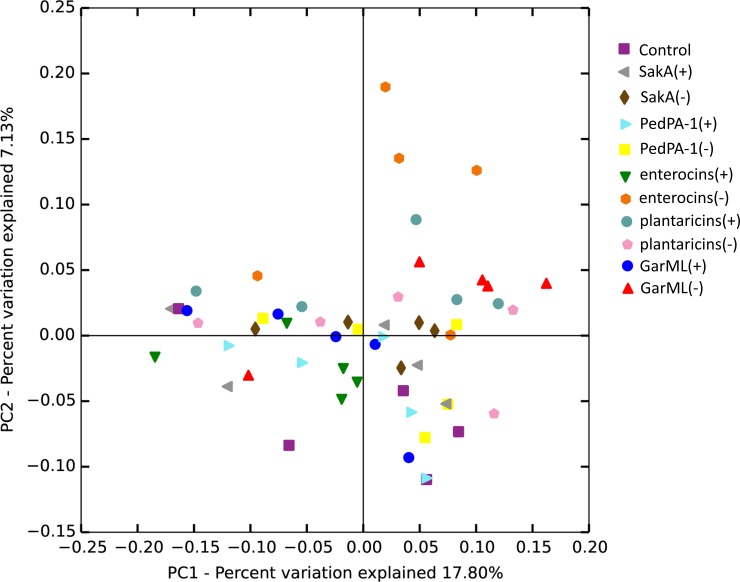
Comparison of bacteria composition of treatments. Principle coordinate analysis (PCoA) plot was generated based on the calculated distances in an unweighted UniFrac matrix. Samples were grouped by color in terms of treatment group they belong to (see legend). (For statistics see S4 Table).

Further, the distance matrices were calculated by unweighted UniFrac and visualized by PCoA plots to compare the communities of bacteriocin-positive, negative and CON cages. In addition to the pooled samples, we also analyzed fecal samples from three randomly selected mice at the same time points (day 0, day 14 and day 28) to examine whether there were any major individual differences compared to the pooled communities. No significant differences were found (S2 Fig, S3 Table). OTU compositions of all the treatment samples were similar to that of the CON at control time point day 0 (i.e., before treatments) as expected (Fig 3), and there was no significant divergence in the community composition over time within the different treatments including CON (S4 Table). Moreover, the OTU compositions were not significantly affected by the different treatments considering all time points (Fig 3 and S4 Table).

These observations together support the general idea that the gut microbiota is relatively resilient in healthy animals (see discussion below).

### Taxa at phylum level

**Fig 4:**
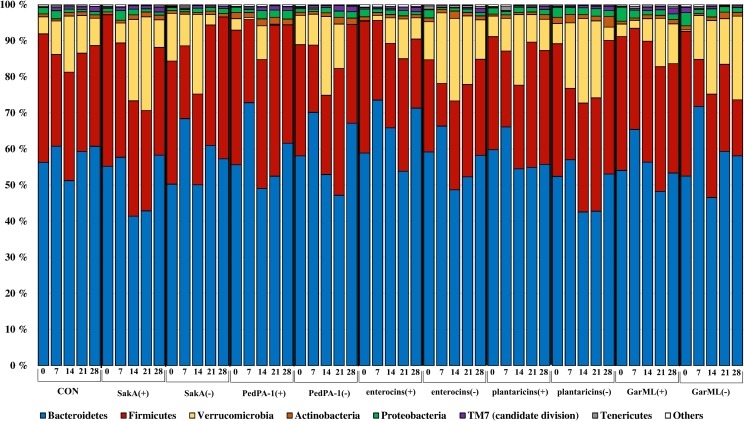
Relative abundances of bacterial phyla in every sample. Different colored bars represent different phyla with size showing relative abundance of this phylum. Labels contain name of treatments and time with day numbers: 0 (day 0), 7 (day 7), 14 (day 14), 21 (day 21) and 28 (day 28).

Similar to the OTU compositions, the relative abundances of bacteria phyla were not strongly affected by bacteriocin producers/non-producers. Overall, the microbiota of mice were dominated by the phyla Bacteroidetes (average of 57% in different treatment groups), Firmicutes (average of 29%) and Verrucomicrobia (average of 10%) (Fig 4). Phyla with a relative abundance of less than 2% were Actinobacteria, Proteobacteria, TM7 (candidate division), Tenericutes and a number of unclassified bacteria. Only a few of the low abundant taxa were predicted to be affected by bacteriocin producers/non-producers. For instance, TM7 (candidate division) decreased in the presence of the SakA(+), SakA(-) and GarML(-) strains, and Proteobacteria by the SakA(+) strain. On the other hand, the Actinobacteria population increased in samples with the SakA(-) treatment compared to CON.

### Changes at lower taxonomic levels

**Fig 5:**
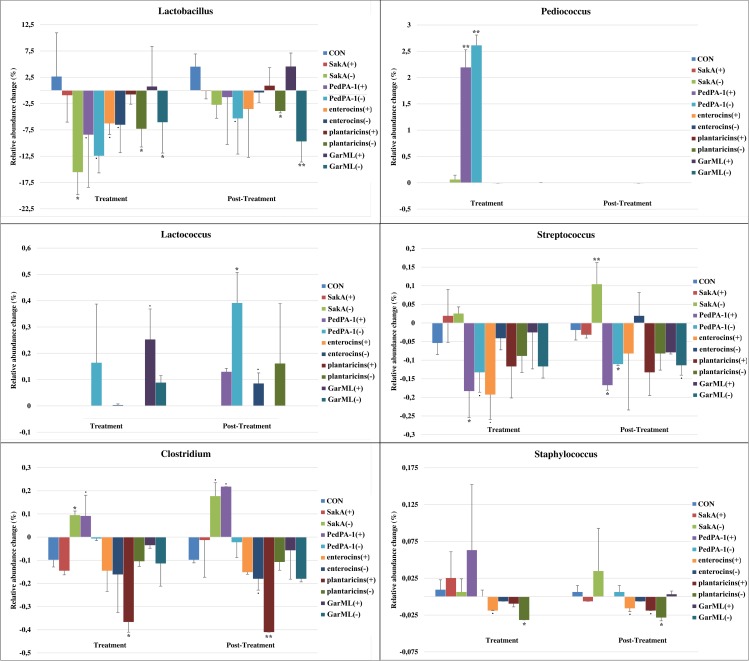
Changes in relative abundance of LAB and bacteriocin-targeted bacterial groups at genus level during treatment and post-treatment periods. Changes in relative abundances of genera in treatments, obtained with respect to time 0, were compared to that in CON. Significance degree is represented as following: P < 0.1 with dot (.); P < 0.05 with one star (*); P < 0.01 with two stars (**).

When analyzing at lower taxonomic levels (i.e., family and genus) we found a number of changes that co-occurred in the presence of bacteriocin producers (Table 2), thus these changes were likely due to bacteriocin effects. Mice that were treated with the producers of SakA and GarML were observed to have increased Leuconostocaceae populations during the treatment period (S3 Fig). The producers of PedPA-1 and plantaricins co-occurred with increases and decreases of the Clostridiaceae population respectively, particularly the genus *Clostridium*, with persistent effects observed during both treatment and post-treatment periods (Fig 5). The Enterococcaceae family population was observed to increase by the producer of enterocins during the treatment period, but this effect disappeared during the post-treatment period (S3 Fig). Unlike enterocins, the producer of GarML was predicted to inhibit the Enterococcaceae family over the entire course of the experiment. The population of Streptococcaceae (particularly *Lactococcus*) was observed to increase in the presence of the GarML producer during the treatment period. On the contrary, this family (particularly *Streptococcus*) became less abundant in mice that were treated with the enterocins producer (Fig 5 and S3 Fig). Moreover, the enterocins producer was predicted to reduce the Staphylococcaceae family, particularly the *Staphylococcus* genus (Fig 5), which is normally associated with various infections and diseases.

**Table 2:** Significant modifications of the relative abundance of LAB and bacteriocin-targeted bacterial groups in response to the treatments.

	SakA	PedPA-1	enterocins	plantaricins	GarML
**Bacteriocin-associated effect**	Leuconostocaceae **↑**	Clostridium ↑*	Enterococcaceae **↑**	Clostridium ↓*	Leuconostocaceae **↑**
			Streptococcus **↓**		Lactococcus **↑**
			Staphylococcus ↓*		Enterococcaceae ↓*
	Total LAB **↑**			Total LAB **↑**	Total LAB **↑**
**Non-bacteriocin-associated effect**		Pediococcus **↑**	Lactobacillus **↓**		
		Lactobacillus **↓**			
		Streptococcus **↓**			
		Enterococcaceae **↓**			

* Bacteriocin-associated modifications that were persistent throughout the experimental period.

In some cases, we also observed similar changes caused by both bacteriocin producers and the isogenic mutants (Table 2). For instance, mice with *P*. *acidilactici* 347 and the isogenic bacteriocin non-producing mutant were observed to have reduced Enterococcaceae (during the treatment period) and *Streptococcus* levels (during the entire course of experiment). Both strains co-occurred with significantly increased *Pediococcus* populations and reduced *Lactobacillus* populations during the treatment period, while the family they are affiliated to, Lactobacillaceae, was not significantly affected. Similarly, *E*. *faecium* L50 and its isogenic mutant L50/14-2 co-occurred with reduced *Lactobacillus*. Moreover, *L*. *plantarum* C11B (bacteriocin producer) during the course of the post-treatment, and its isogenic mutant strain *L*. *plantarum* C11D3 during the whole experiment, showed a significant adverse effect on the Staphylococcaceae population (S3 Fig).

It is well known that Gram-negative bacteria as well as Gram-positive bacteria that are more distantly related to the bacteriocin producers are generally not sensitive to LAB bacteriocins. We observed that some populations of these bacterial groups were affected, e.g., Firmicutes affiliated-populations: Erysipelotrichaceae, Lachnospiraceae and Ruminococcaceae. However, most of these changes were likely not due to bacteriocin production as both the bacteriocin producers and their isogenic mutants gave similar effects (S5 Table). Only the GarML producer, but not the isogenic strain, co-occurred with increased Ruminococcaceae populations in the post-treatment period (S5 Table).

Surprisingly, some bacteriocin producers and their isogenic mutants behaved differently toward the Gram-negative bacteria (S5 Table). The Bacteroidaceae population was observed to increase in the presence of the SakA producing strain during the treatment period but the isogenic mutant (bacteriocin negative) during the post-treatment period. The Prevotellaceae and Rikenellaceae populations were significantly more abundant in the samples treated with the producer of GarML compared to CON samples, while no change was observed in those treated with the isogenic strain of GarML. Nevertheless, there were also cases where both the bacteriocin producers and their isogenic mutants were predicted to cause the same changes (S5 Table). F16 (affiliated to TM7 candidate division) levels were reduced in the presence of both the producer and non-producer strains of plantaricins, while Desulfovibrionaceae, which is a subgroup of Proteobacteria, was increased in mice fed by both the producers and non-producers of SakA and PedPA-1.

### Blood serum components

**Fig 6:**
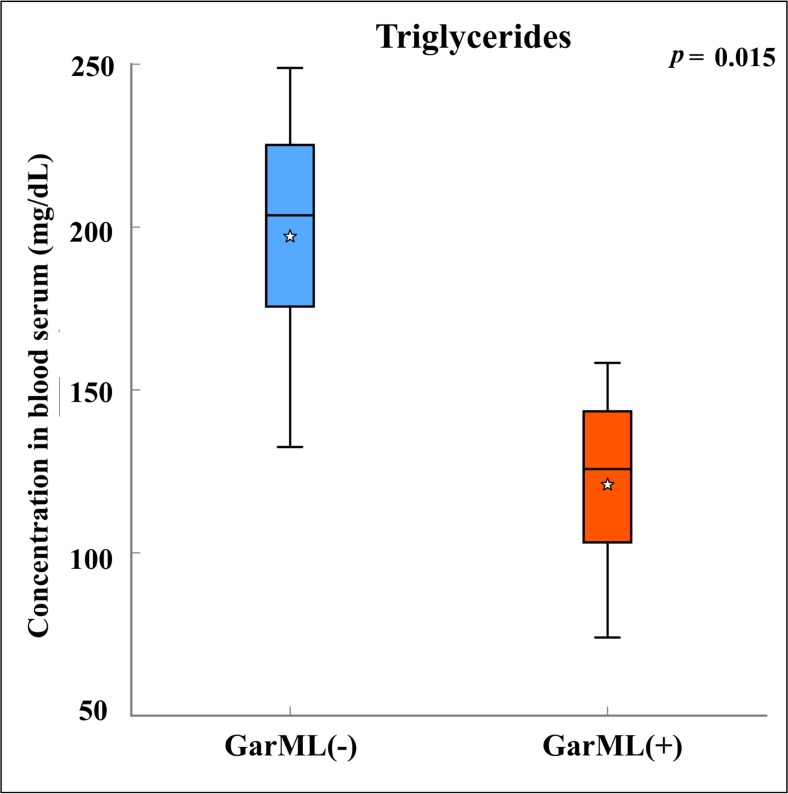
Significant serum level modifications by Garvicin ML. The pairwise comparisons between bacteriocin positive and negative treatments were performed using Student’s t-test. The boxplot shows the significant comparison with *P* < 0.05. GarML: Garvicin ML, (+): bacteriocin producing strain, (-): bacteriocin non-producing isogenic strain treatments.

Next, we focused on the blood serum components at the end of the treatment period, i.e., the levels of triglycerides, total cholesterol, HDL and LDL, which can be used to estimate the risk of some health disorders, such as heart diseases and obesity. A significant decrease in the level of triglycerides, which is considered beneficial to host, was observed in mice treated with GarML(+), compared to bacteriocin negative treatment (Fig 6).

**Fig 7:**
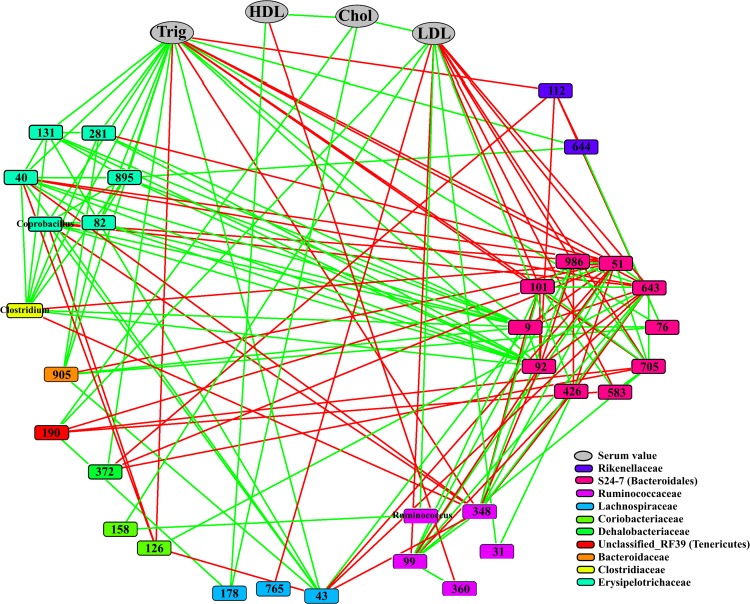
Correlation network of relative abundances of OTUs at day 14 and serum levels. The correlations were calculated using Pearson’s correlation in CoNet and the significant ones (P < 0.05) were shown on the network. All serum values are shown by one color (grey) while OTUs belonging to different families are represented by different colors (see legend). Positive correlations are displayed with green edges and negative correlations with red edges. OTUs on the nodes were represented with OTU numbers or genus names they belong to and serum values were labelled as Trig: triglycerides, HDL: high-density lipoprotein, Chol: total cholesterol and LDL: low-density lipoprotein.

Correlations between OTUs and the level of the blood serum components were also investigated. Significant correlations were found for some OTUs of the families Rikenellaceae, S24-7 (Bacteroidales subgroup), Ruminococcaceae, Lachnospiraceae, Coriobacteriaceae, Dehalobacteriaceae, Unclassified RF39 (Tenericutes subgroup), Bacteroidaceae, Clostridiaceae and Erysipelotrichaceae (*P* < 0.05) (Fig 7). The most remarkable correlations were found with OTUs affiliated to the S24-7. They were mostly negatively correlated with serum LDL and triglycerides levels. On the other hand, the OTUs of Erysipelotrichaceae family were positively correlated with the levels of triglycerides. The Bacteroidaceae population was also positively correlated with the level of triglycerides, while one OTU belonging to Rikenellaceae showed a negative correlation. Moreover, the correlation of Ruminococcaceae with the measured serum levels was more OTU-specific.

## Discussion

Production of bacteriocins by LAB has generally been considered as a potential probiotic trait. The target specificity of the bacteriocins against a number of pathogenic or antibiotic-resistant bacterial strains has been of great interest in many research groups [20–25]. However, only a few studies have assessed the *in situ* effect of bacteriocin producing LAB on the normal gut microbiota in live animals. For example, *L*. *salivarius* UCC118 producing bacteriocin Abp118 has been shown to cause significant but subtle changes on the murine and pig intestinal microbiota [49]. The impact of the combination of probiotics including one bacteriocin-producing strain (a *L*. *salivarius* strain producing salivaricin P) was evaluated in pig model and the predominance of the bacteriocin producing strain has been observed [50]. Another study showed a shift in the fecal bacterial structure in humans caused by the probiotic strain, *L*. *plantarum* P-8, and this shift has been suggested to be due to the putative plantaricin production by this strain [51]. Nevertheless, all these studies have assessed only one bacteriocin producer at a time and most of the times without an isogenic bacteriocin-negative strain as control. The lack of such control strains makes it difficult to attribute the observed changes to bacteriocin production itself or to other unknown non-bacteriocin activities.

In our study, LAB producers of various class II bacteriocins (i.e., SakA, PedPA-1, enterocins (Q and L50), plantaricins (EF and JK) and GarML) were assessed for potential probiotic properties in healthy mice. All these producers were accompanied with a set of corresponding isogenic strains whose bacteriocin production is deleted or reduced, to serve as reference strains. The bacteriocins produced are categorized into different subclasses with different physicochemical properties, structures and antimicrobial spectra (S6 Table) [52], thus allowing us to study how different bacteriocins affect the gut microbiota. Moreover, the samples were collected during both the treatment period and the post-treatment period so that we could evaluate the persistence of the effects over time. Some of the bacteriocins used herein have antimicrobial activity against pathogenic strains at least *in vitro*, for example, SakA and PedPA-1 against *Listeria* spp., enterocins against *S*. *aureus* (S6 Table) and GarML against several pathogens including *Listeria* and *Clostridium* spp. [32].

In general, our results indicate that the main structure of the gut bacterial composition in mice was relatively resilient to the administration of LAB producers or non-producers. However, at lower taxonomic levels (at genus level) we could observe some modifications and these changes varied in a manner related to the *in vitro* antimicrobial activities of the bacteriocins, i.e., the narrow-spectrum bacteriocins SakA, PedPA-1 and plantaricins showing less impact on bacterial communities compared to the wider-spectrum bacteriocins enterocins and GarML.

The populations of Gram-positive bacteria, particularly LAB such as lactobacilli, lactococci, enterococci, streptococci, leuconostoc, and pediococci as well as some other niche competing bacteria like staphylococci, listeria and clostridia are often the targets of LAB-produced bacteriocins [53]. As expected, these populations were altered mostly when mice were treated with bacteriocin producers. Some of these changes can be favorable to the host, such as the reduction in population size of streptococci, staphylococci, and clostridia. However, it should be underlined that pathogenicity of an organism can vary greatly between species within a genus, or even between strains within a species. For instance, some members of clostridia are known to be beneficial for the host contributing to the maintenance of the gut homeostasis [54], especially for ruminants (polygastric hosts) improving digestion of complex organic matter such as cellulose [55]. On the other hand, some species of this genus are often associated with diverse infections and diseases in monogastric animals, as well as being regarded as opportunists in humans (e.g., *C*. *perfringens*) [56]. The same is true for the *Enterococcus faecalis* and *Escherichia coli* species, which include probiotic strains, as well as renown pathogenic strains implicated in serious diseases [57]. Some examples of the pathogenic members of the aforementioned bacterial groups are *Streptococcus bovis* [58], *S*. *aureus* [59], *C*. *difficile* [56]. However, due to the limited resolution of 16S rRNA gene sequencing, the species-level identification of these bacterial groups could not be conducted in this study.

In addition to modification of specific taxa, some bacteriocin producers (SakA, plantaricins and GarML) co-occurred with significantly increased the counts of total LAB. This is interesting since LAB can have probiotic value via the production of various molecules (e.g., short chain fatty acids, conjugated linoleic acids, exopolysaccharides, fructooligosaccharides and selenoproteins) and favor the production of butyrate and propionate of other mutualistic bacteria [60]. LAB are also involved in regulating host metabolism and immune system, controlling infections and modulating inflammation [61].

In spite of several direct correlations, some of the *in vitro* antimicrobial activities of bacteriocins were not observed in the gut communities in mice. For example, the class IIa bacteriocins, SakA and PedPA-1, were seemingly effective against *Enterococcus* strains and GarML against *Lactobacillus* strains *in vitro*; however, these bacteriocins did not inhibit the growth of these populations *in vivo*. These discrepancies between *in vitro* and *in vivo* effects are not surprising because several factors can affect bacteriocin production *in vivo* and even the bacteriocin activity. For example, the bacteriocin production can be regulated by cues from the environment, such as signal transduction systems (quorum-sensing) known to be involved in SakA and plantaricins biosynthesis, and temperature-dependent regulation in enterocins biosynthesis [33,62–65]. In addition, niche competition between bacteria, other antimicrobial compounds that are produced by LAB (e.g., organic acids, hydrogen peroxide, antifungal peptides) and the host responses can influence the environment [66,67].

The fecal populations of *Enterococcus* and *Lactococcus* increased in cages treated with the enterococcal enterocins and the lactococcal GarML, respectively. It is likely that a significant proportion of the reads corresponding to these bacterial groups would correspond to the bacteriocin producers themselves and that their colonization in gut was facilitated by bacteriocin production. This later notion is supported by the fact that the corresponding isogenic mutants did not result in an increase of these populations. However, the *Pediococcus* population was enhanced in the presence of both the PedPA-1 producer and its isogenic mutant; therefore, this enhancement could not be linked only to the bacteriocin production. Moreover, there was no obvious increase in the *Lactobacillus* population in cages treated with SakA or plantaricins (bacteriocins produced by lactobacilli). The detection of changes in the relative abundance of this population was challenging due to the large number of affiliated OTUs. However, it is likely that the growth of native *Lactobacillus* strains was triggered or they were replaced by the administrated bacteriocin-producing *Lactobacillus* strains as suggested before [25].

Bacteriocins may also help the producer invade a new niche by competitive exclusion of other inhabitants, which usually are closely related bacteria, such as LAB in this study. This competition can lead to modifications of other bacterial populations connected in the microbial network [25]. This might explain the increase of Prevotellaceae in the presence of plantaricins and GarML, and the effect of GarML on Ruminococcaceae and other Bacteroidetes affiliated bacteria (e.g., Rikenellaceae). Members of these Gram-negative families are known to produce short chain fatty acids beneficial for host [68–70]. Moreover, we observed that the populations of Bacteroidetes phylotypes S24-7, Bacteroidaceae, Rikenellaceae, Ruminococcaceae, Erysipelotrichaceae, Coriobacteriaceae, Lachnospiraceae and *Clostridium* were correlated in an OTU-specific manner with the serum parameters.

Weight gain is one of the parameters to measure host health. The treated mice gained weight in a manner comparable to or better than the CON cage, indicating that the bacteriocin/bacteria treatments did not have a negative impact on the normal growth of mice. The producer of PedPA-1 significantly increased the body weight of mice compared to CON, in contrast to its corresponding isogenic mutant. On the other hand, both producer and its isogenic non-producer of GarML and enterocins had a positive impact on weight in the treatment period (GarML isogenic strain also in the post-treatment period), implying that the effects were seemingly due to the strains, independently of the bacteriocin phenotype. Variation in weight gain in response to administration of bacteriocins or certain strains has been observed before [49,71]. Blood serum levels of triglycerides were significantly decreased by the producer of GarML. This change is potentially positive for the host because the blood level of triglycerides is a physiological indicator of the risk of some health disorders such as heart diseases and obesity. However, it is of future interest to correlate the effects of bacteriocins on host physiology to gut microbiota modifications.

The gut environment is a complex niche where numerous and diverse bacteria are thriving and competing fiercely for common resources. The successful survivors must therefore have developed strategies to coexist with other gut inhabitants and with the host in an interactive network. This network is presumably quite resilient as well as dynamic in order to deal with many different chemical challenges during the daily traffic along the intestinal tract (e.g., ingested food or medicines) and to maintain the diverse functions the gut play (processing the ingested food, producing nutrients, vitamins, immune-stimulation, gut emptying, etc.). Such a resilient and dynamic nature of the healthy gut can be seen in our present study. Firstly, the overall structure of microbiota remained largely unaffected by the administration of bacteriocin producers and non-producers of different genera. Secondly, significant changes were observed at lower taxonomic levels in the gut of treated mice, depending on the bacteriocin produced. However, many of these changes disappeared or were disappearing at the end of the 4-week course of experiment. In terms of probiotic use, these properties are highly appreciated because probiotics are meant to transiently affect the gut microbiota to promote health-bringing conditions for host (inhibition of potential pathogens, enhanced growth of beneficial bacteria, increase of beneficial blood serum parameters, etc.) without disturbing the gut’s main microbial structure and function.

Nevertheless, although probiotics have been much studied worldwide in recent years and there are numerous studies showing different probiotic effects from LAB and other bacteria, this research field is still, at best, in its infancy, especially with regard to the limited understanding at the molecular and cellular levels. Further, it is important to underline that the use of probiotics to achieve favorable values in animals is a rather complex and unpredictable process as many unknown hurdles along the GIT can deteriorate or repress the probiotic properties. Some probiotic properties can even vary dependently on a number of host parameters, including animals tested, genetic background, age and gender [72]. Thus, to fully and safely appreciate their health bringing values, probiotics must be critically and carefully assessed in the relevant models and settings.

## Supporting Information

S1 Fig Relative abundances of OTUs at genus level. Different colored bars represent different genera with size showing relative abundance of this genus. Labels contain name of treatments and time with day numbers: 0 (day 0), 7 (day 7), 14 (day 14), 21 (day 21) and 28 (day 28).
(TIF)

S2 Fig Comparison of bacterial composition of pooled fecal samples and fecal samples from individual mice in CON cage. Principle coordinate analysis (PCoA) plot was generated based on the calculated distances in an unweighted UniFrac matrix. Samples were grouped by color and shape such that pool of fecal samples at indicated time point (red circle), day 0 (T = 0) individual mice samples (blue square), day 14 individual mice samples (green square) and day 28 individual mice samples (orange square). Individual mice were indicated with numbers: M5, M7 and M10. (For statistics see S3 Table).
(TIF)

S3 Fig Changes in relative abundance of LAB and bacteriocin-targeted bacterial groups at family level during treatment and post-treatment periods. Change in relative abundances of families corresponding to day 0 of treatments were compared to CON. Significance degree is represented as followings: P<0.1 with dot (.); P<0.05 with one star (*); P<0.01 with two stars (**).
(TIF)

S1 Table Lactic acid bacteria count in treatment samples. *The log values in bacteriocin positive and negative treatments were compared pairwise using two-sided Student's t-test for each bacteriocin group.
(XLSX)

S2 Table Shannon indexes calculated as mean of ten iterations at equal subsampling size of 15,840. (XLSX)

S3 Table Comparison of UniFrac distances of pooled and individual samples. The statistical significances of differences in unweighted UniFrac distances in terms of groups (pooled vs individual samples) and time were shown. The non-parametric p-values were calculated performing two-sample t-tests for the pairs of the groups with Monte Carlo permutations (n = 1,000) and corrected with Bonferroni method.
(XLSX)

S4 Table Comparison of UniFrac distances between treatments. The statistical significances of differences in unweighted UniFrac distances between treatments and time points were shown. The treatments that were compared for each bacteriocin group included control, bacteriocin positive and bacteriocin negative treatments. The nonparametric p-values were calculated performing two-sample t-tests for the pairs of the groups with Monte Carlo permutations (n = 1,000) and corrected with Bonferroni method.
(XLSX)

S5 Table Comparison of relative abundance change of families by administrated strains compared to CON in treatment and post-treatment periods. The difference of the relative abundances from time 0 within each treatment was calculated. The changes at day 7 and day 14 were analyzed as “treatment”, while day 21 and day 28 were analyzed as “post-treatment” data. ANOVA was performed on the relative abundance changes in control, bacteriocin-producer and bacteriocin-non-producer groups. '(+)' indicates increase and '(-)' indicates decrease compared to CON and significance degree is represented as followings: P<0.1 with dot (.); P<0.05 with one star (*); P<0.01 with two stars (**).
(XLSX)

S6 Table In vitro antimicrobial activity assay of bacteriocins against the indicator strains listed. (XLSX)

## Abbreviations

LAB: lactic acid bacteria
HDL: high-density lipoprotein
LDL: low-density lipoprotein
CON: control with no treatment
SakA: sakacin A
PedPA-1: pediocin PA-1
enterocins: enterocins Q and L50
plantaricins: plantaricins EF and JK
GarML: garvicin ML
maxee: maximum expected error
OTU: operational taxonomic unit
RDP: Ribosomal Database Project
PCoA: Principle coordinate analysis
