# Supplementary figures and images for: The Potential of Class II Bacteriocins to Modify Gut Microbiota to Improve Host Health
Source: PLoS One. 2016 Oct 3;11(10):e0164036. doi: 10.1371/journal.pone.0164036 (PMC5047636; doi:10.1371/journal.pone.0164036)

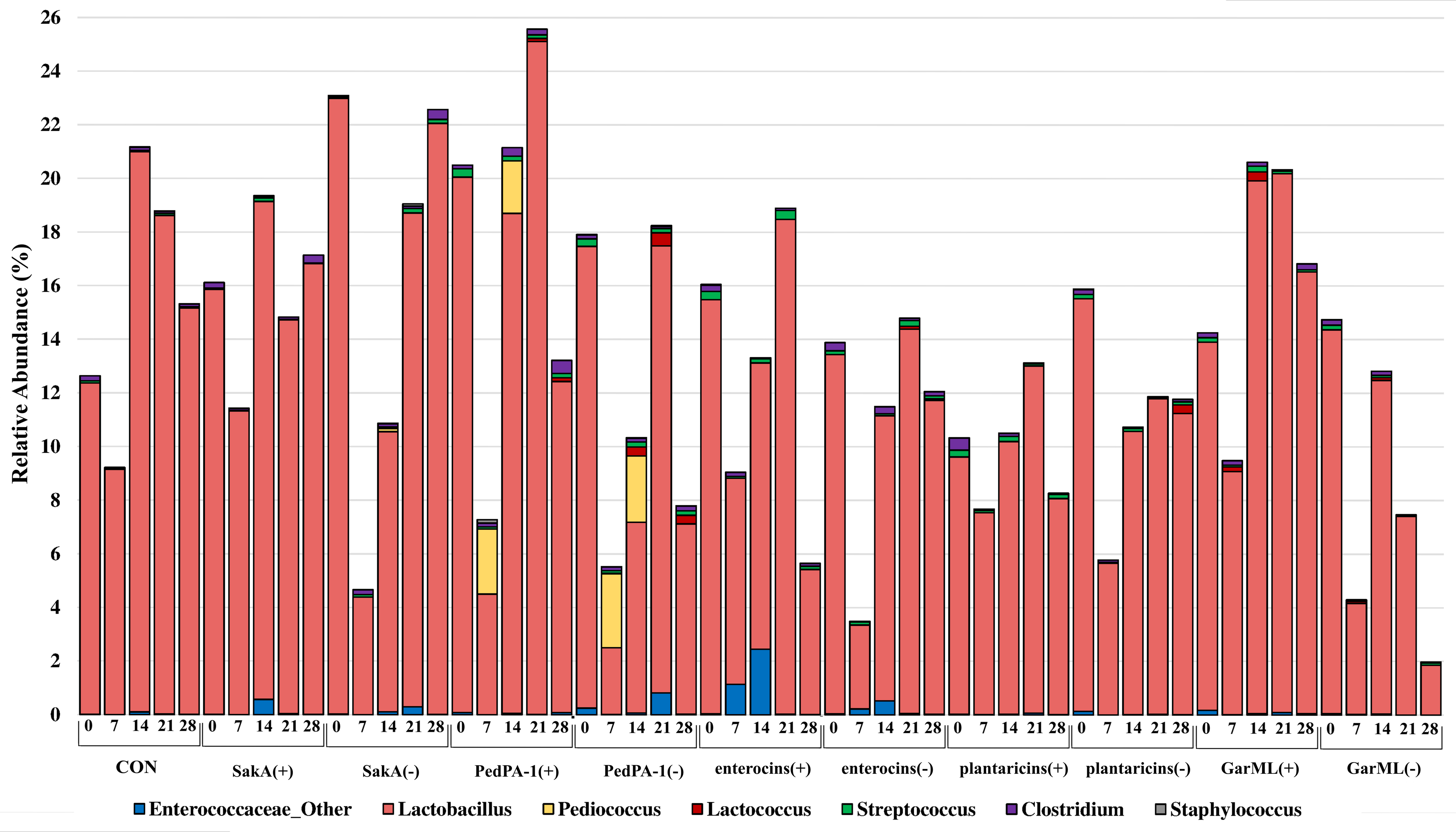

Supplement: S1 Fig — Different colored bars represent different genera with size showing relative abundance of this genus. Labels contain name of treatments and time with day numbers: 0 (day 0), 7 (day 7), 14 (day 14), 21 (day 21) and 28 (day 28). (TIF) [file pone.0164036.s001.tif]

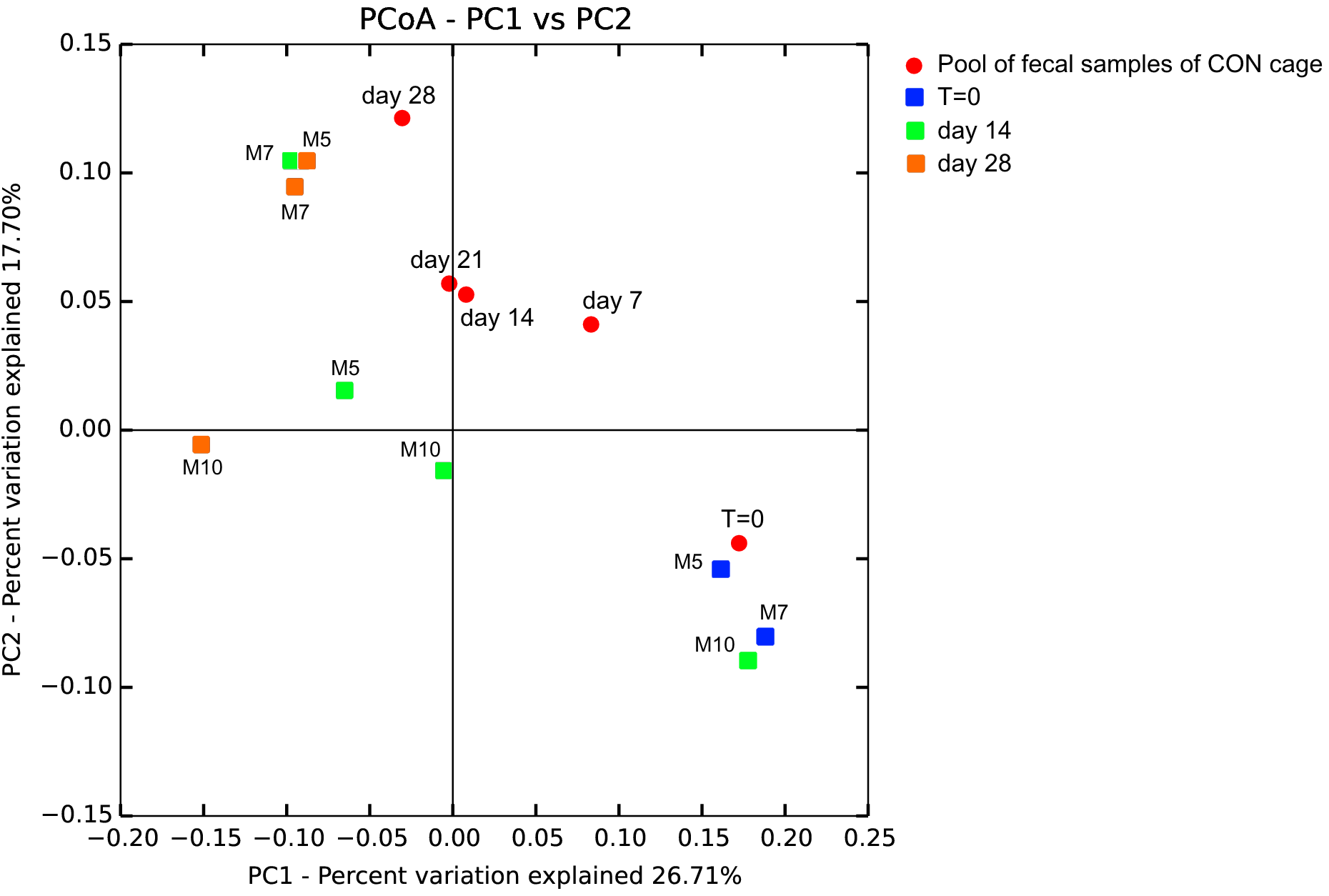

Supplement: S2 Fig — Principle coordinate analysis (PCoA) plot was generated based on the calculated distances in an unweighted UniFrac matrix. Samples were grouped by color and shape such that pool of fecal samples at indicated time point (red circle), day 0 (T = 0) individual mice samples (blue square), day 14 individual mice samples (green square) and day 28 individual mice samples (orange square). Individual mice were indicated with numbers: M5, M7 and M10. (For statistics see S3 Table). (TIF) [file pone.0164036.s002.tif]

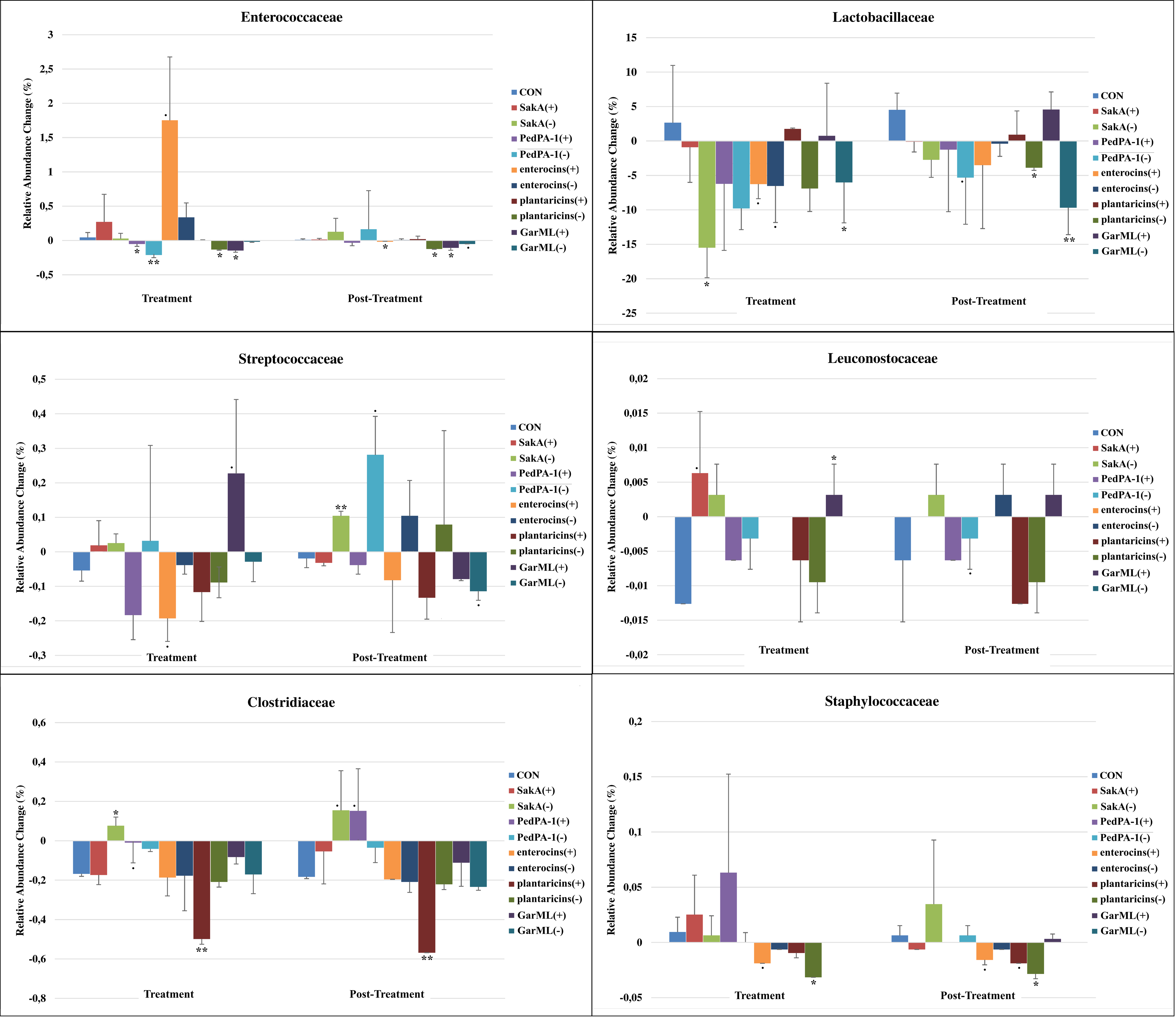

Supplement: S3 Fig — Change in relative abundances of families corresponding to day 0 of treatments were compared to CON. Significance degree is represented as followings: P<0.1 with dot (.); P<0.05 with one star (*); P<0.01 with two stars (**). (TIF) [file pone.0164036.s003.tif]
